# E8002 Inhibits Peripheral Nerve Adhesion by Enhancing Fibrinolysis of l-Ascorbic Acid in a Rat Sciatic Nerve Model

**DOI:** 10.3390/ijms21113972

**Published:** 2020-06-01

**Authors:** Kiyoshi Kikuchi, Kentaro Setoyama, Seiya Takada, Shotaro Otsuka, Kazuki Nakanishi, Kosuke Norimatsu, Akira Tani, Harutoshi Sakakima, Ko-ichi Kawahara, Kazuya Hosokawa, Ryoji Kiyama, Megumi Sumizono, Salunya Tancharoen, Ikuro Maruyama, Gohsuke Hattori, Motohiro Morioka, Eiichiro Tanaka, Hisaaki Uchikado

**Affiliations:** 1 Division of Brain Science, Department of Physiology, Kurume University School of Medicine, Kurume, Fukuoka 830-0011, Japan; kikuchi_kiyoshi@kurume-u.ac.jp; 2 Department of Neurosurgery, Kurume University School of Medicine, Kurume, Fukuoka 830-0011, Japan; hattori_gohsuke@kurume-u.ac.jp (G.H.); mmorioka@med.kurume-u.ac.jp (M.M.); 3 Department of Systems Biology in Thromboregulation, Kagoshima University Graduate School of Medical and Dental Science, Kagoshima 890-8520, Japan; k5082701@kadai.jp (S.T.); k3360022@kadai.jp (S.O.); koichi.kawahara@oit.ac.jp (K.-i.K.); maruyama@m2.kufm.kagoshima-u.ac.jp (I.M.); 4 Department of Pharmacology, Faculty of Dentistry, Mahidol University, Bangkok 10400, Thailand; salunya.tan@mahidol.edu; 5 Natural Science Center for Research and Education, Division of Laboratory Animal Science, Kagoshima University, Kagoshima 890-8520, Japan; seto@m.kufm.kagoshima-u.ac.jp; 6 Department of Physical Therapy, School of Health Sciences, Faculty of Medicine, Kagoshima University, Kagoshima 890-8544, Japan; k9378361@kadai.jp (K.N.); k6745961@kadai.jp (K.N.); k3694885@kadai.jp (A.T.); sakaki@health.nop.kagoshima-u.ac.jp (H.S.); kiyama@health.nop.kagoshima-u.ac.jp (R.K.); 7 Laboratory of Functional Foods, Department of Biomedical Engineering, Osaka Institute of Technology, Osaka 535-8585, Japan; 8 Research Institute, Fujimori Kogyo Co., Ltd., 1-10-1 Sachiura, Kanazawa-ku, Yokohama, Kanagawa 236-0003, Japan; kazuya-hosokawa@zacros.co.jp; 9 Department of Rehabilitation, Faculty of Nursing and Welfare, Kyushu University of Nursing and Social Welfare, Tamana, Kumamoto 865-0062, Japan; sumizono@kyushu-ns.ac.jp; 10 Uchikado Neuro-Spine Clinic, Fukuoka 812-0893, Japan

**Keywords:** peripheral nerve adhesion, anti-adhesive membrane, E8002, l-ascorbic acid, antioxidant

## Abstract

Perineural adhesions leading to neuropathy are one of the most undesirable consequences of peripheral nerve surgery. However, there are currently no widely used compounds with anti-adhesive effects in the field of peripheral nerve surgery. E8002 is a novel, anti-adhesive, multi-layer membrane that contains L-ascorbic acid (AA). Here, we investigated the effect and mechanism of E8002 in a rat sciatic nerve adhesion model. A total of 21 rats were used. Six weeks after surgery, macroscopic adhesion scores were significantly lower in the E8002 group (adhesion procedure followed by nerve wrapping with E8002) compared to the E8002 AA(−) group (adhesion procedure followed by nerve wrapping with the E8002 membrane excluding AA) and adhesion group (adhesion procedure but no treatment). Correspondingly, a microscopic examination revealed prominent scar tissue in the E8002 AA(−) and adhesion groups. Furthermore, an in vitro study using human blood samples showed that AA enhanced tissue-type, plasminogen activator-mediated fibrinolysis. Altogether, these results suggest that E8002 may exert an anti-adhesive action via AA and the regulation of fibrinolysis.

## 1. Introduction

The neurolysis of the peripheral nerves is an effective and common surgical procedure for entrapment neuropathy [1]. However, it always results in scar tissue due to the infiltration of fibroblasts and myofibroblasts. In turn, extraneural and intraneural scar formation causes peripheral nerve adhesions [1]. Moreover, clinical symptoms recur in most patients because of the re-appearance of secondary perineural adhesions and neural fibrosis, which result in impaired nerve function [2]. To prevent the formation of perineural adhesions, several therapeutic approaches, including implantation of muscle flaps, fat grafts, and grafts using diverse biomaterials, have been tested. Nevertheless, most therapies remain experimental [3], and none are routinely used on patients. Indeed, within the field of peripheral nerve surgery, there are currently no compounds with anti-adhesive effects that are in widespread use globally. Consequently, the issues of postoperative adhesion and scar problems have not been resolved. One previously used approach was to separate tissues that are likely to adhere by using a physical barrier [4]. However, the current lack of anti-adhesive methods in the clinical setting suggests that a physical barrier alone may not completely prevent adhesions. A new approach is therefore necessary.

Fibrous adhesion formation is regulated by the balance between tissue-type plasminogen activator (tPA) and plasminogen activator inhibitor type 1 (PAI-1), which reciprocally regulate fibrin deposition [5]. Thus, tPA and PAI-1 are possible therapeutic targets for preventing postoperative adhesion formation via fibrinolysis. Relatedly, oxidative stress is a key contributor of adhesion formation [6,7], which may also act via the fibrinolytic system and the reciprocal balance between tPA and PAI-1 [5]. Accordingly, the fibrin-binding affinity of tPA can be impaired by exposure to oxidative stress [8]. Recently, we reported that antioxidants, such as uric acid and edaravone, enhance fibrinolysis [9,10]. Furthermore, in a rat uterine horn model, the intraperitoneal administration of antioxidants, such as vitamin C (l-ascorbic acid (AA)) or vitamin E, effectively prevented postoperative adhesion formation [11]. Thus, although the mechanism underlying the anti-adhesive effect of antioxidants is unclear, our study showed that this molecular mechanism might also be involved in adhesion formation after peripheral nerve surgery.

**Figure 1:**
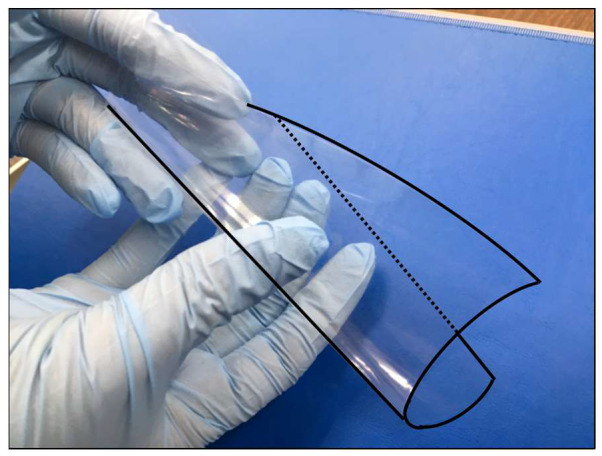
E8002 membrane. E8002 is a flexible three-layered membrane comprising a polylactic acid-based biodegradable polymer containing the antioxidant l-ascorbic acid. For image clarity, the outline of the membrane has been highlighted using a solid and dotted line.

We previously developed a polylactic acid-based biodegradable polymer named E8002 (Figure 1) [12]. E8002 has a three-layered structure: the central layer comprises bioabsorbable pullulans (polysaccharide polymers used in foods and drugs), while the surface layers comprise l-lactide, glycolide, and ε-caprolactone copolymers, which are also used in bioabsorbable sutures. The central layer is 30–50 µm thick and readily dissolves under moist conditions, while the surface layers are approximately 100 nm thick and nearly insoluble. l-ascorbic acid is contained in both the central and surface layers. We recently reported that E8002 can reduce adhesions in a rat cecal adhesion model and a rat laminectomy model [12,13]. Because E8002 contains the antioxidant AA, we wanted to determine if AA mediates the anti-adhesive effect of E8002. To do this, we examined the effect and underlying mechanism of E8002 with or without AA by using a rat sciatic nerve adhesion model. We chose to use this model because it provides a wider surgical field than the rat laminectomy model. Furthermore, we suspect that E8002 has anti-adhesive effects in various types of surgery, a fact that can be corroborated using alternative animal models. Finally, to provide clinical relevance, we also examined the effect of AA in an in vitro model of thrombolysis using human blood samples.

## 2. Results

### 2.1. Wound Healing Is Not Affected by E8002 Membrane

**Figure 2:**
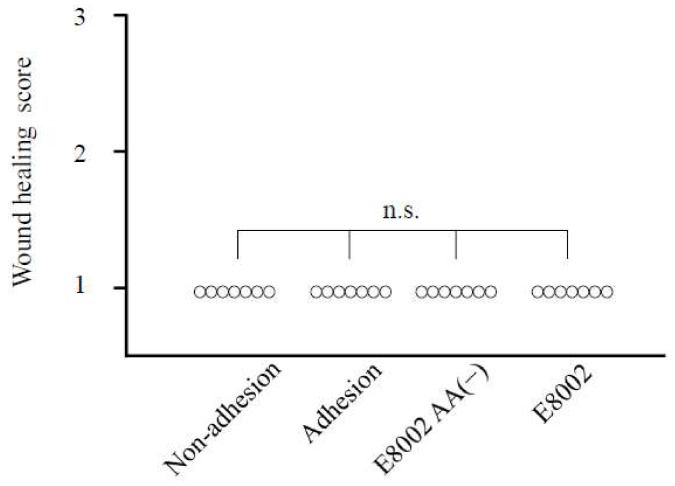
Effect of the E8002 membrane on wound healing score at 6 weeks post-surgery. There were no significant differences in wound healing scores between the three groups (*n* = 7 rats per group). n.s., non-significant as determined by the Kruskal–Wallis test.

Following sham or treatment surgery in the sciatic nerve adhesion model, there were no significant differences in wound healing scores between the three groups (*p* < 0.05; Figure 2). This indicates that both the superficial layer and fascia healed without damage in all groups.

### 2.2. l-Ascorbic Acid-Containing E8002 Membrane Inhibits Formation of Nerve Adhesions

**Figure 3:**
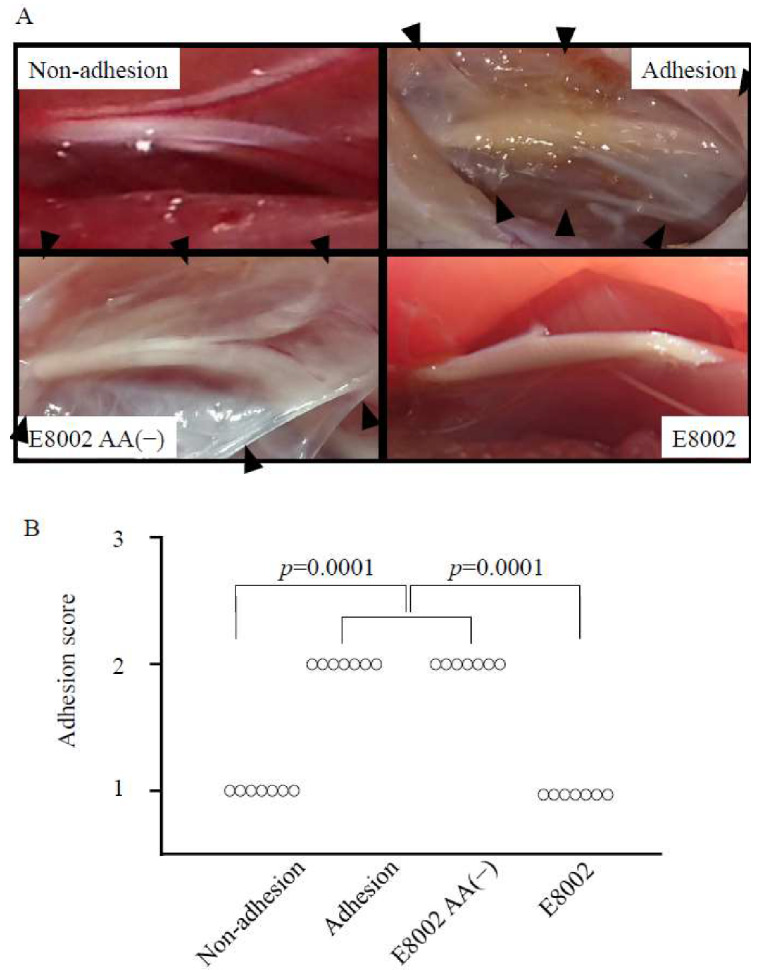
Effect of E8002 membrane on macroscopic peripheral nerve adhesions at 6 weeks post-surgery. (**A**) Representative photomicrographs of the macroscopic appearance of the adhesion site (adhesion, E8002(−), and E8002 groups) or related area (non-adhesion group). The tissue located in the center of each figure is the nerve tissue. The adhesion area is recognizable as fibrous connective tissue located around the nerve tissue (the white area indicated by black arrowheads). (**B**) Adhesion scores in the non-adhesion group and the E8002 group were significantly lower than those in the adhesion group and the E8002 l-ascorbic acid (AA(−)) group (*n* = 7 adhesion sites or related areas per group). *p* = 0.001 as determined by the Kruskal–Wallis test.

Representative images of the gross appearance of adherent nerves at six weeks post-surgery are shown in Figure 3A. In both the adhesion and E8002 AA(−) groups, fibrous connective tissue covered the nerve. Macroscopic adhesion scores were significantly lower in the non-adhesion group (adhesion score, 1 ± 0) and the E8002 group (1 ± 0) than the adhesion group (2 ± 0) and the E8002 AA(−) group (2 ± 0) (*p* = 0.0001, *H* = 20.2758; Figure 3A). This suggests that adhesion formation was reduced following the surgical application of an AA-containing E8002 membrane to the sciatic nerve.

### 2.3. l-Ascorbic Acid-Containing E8002 Membrane Inhibits Scar Tissue Formation

**Figure 4:**
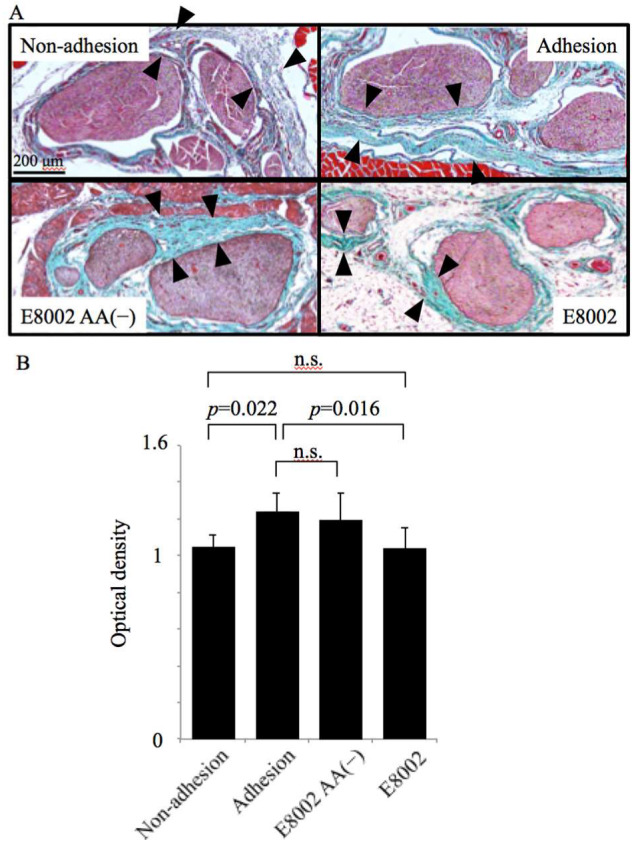
Effect of the E8002 membrane on microscopic appearance of peripheral nerve adhesions at 6 weeks post-surgery. (**A**) Representative photomicrographs of the sciatic nerve and neural bed stained with aldehyde fuchsin Masson–Goldner staining (scale bar, 200 µm). Pink-stained nerve tissue located in the center is surrounded by connective tissue (light blue/green area indicated by black arrowheads). In the E8002 group, there was thin and loose connective tissue around the nerve, with space (no staining) surrounding the connective tissue. This space was considered to be a trace of E8002 absorption. (**B**) Quantitative analysis of the optical density of the Masson–Goldner staining (*n* = 7 adhesion sites or related areas per group). Values are mean ± SE; n.s., non-significant determined by one-way analysis of variance followed by the Bonferroni–Dunn correction.

To determine the extent of scar tissue formation, we examined connective tissue using aldehyde fuchsin Masson–Goldner staining. Representative images of the microscopic appearance of adhesion sites are shown (Figure 4A). Aldehyde fuchsin Masson–Goldner-stained scar tissue revealed thick and dense connective tissue around the nerves in both the adhesion and E8002 AA(−) groups. Notably, scar tissue surrounding the nerve had strongly adhered to the nerve. In comparison, in the E8002 group, although scar tissue was detected between the nerve conduit and neural bed, little perineural scar formation was present around the nerve.

To quantitate the detected scar tissue, we determined the optical density of Masson–Goldner staining (Figure 4B). The optical density was significantly higher in the adhesion group (1.243 ± 0.038) than in the non-adhesion group (1.048 ± 0.027) (*p* = 0.022) and the E8002 group (1.040 ± 0.044) (*p* = 0.016). Moreover, the optical density was comparable in the non-adhesion and E8002 groups, as well as the adhesion and E8002 AA(−) groups (1.194 ± 0.056). Though aldehyde fuchsin Masson–Goldner staining could not distinguish between scar tissue and normal connective tissue, these results suggested that scar tissue formation was reduced following treatment with the AA-containing E8002 membrane.

### 2.4. E8002 Membrane Did Not Cause Neurological Adverse Effects in Rats Following Sciatic Nerve Adhesion

**Figure 5:**
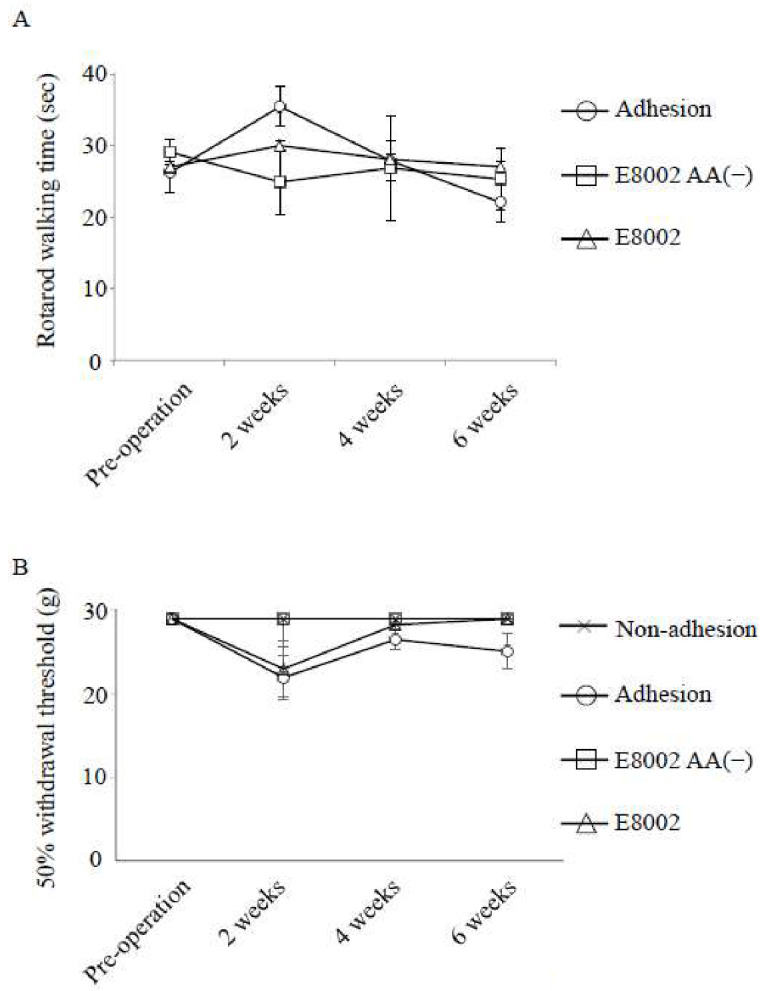
Effect of the E8002 membrane on neurological function. (**A**) Motor function was examined by rotarod walking time (s) (*n* = 7 rats per group). There are no data for the non-adhesion group because this group comprised the healthy limbs of a randomly selected subset of the total rats used. Values are mean ± SE; n.s., non-significant determined by an ANOVA or the Kruskal–Wallis test followed by the Bonferroni–Dunn correction. Time course was analyzed by a repeated measures ANOVA or the Friedman test. (**B**) Mechanical sensitivity determined by the 50% withdrawal threshold (g) (*n* = 7 hindlimbs per group). After surgery, the adhesion group developed mechanical hypersensitivity. Values are mean ± SE; n.s., non-significant determined by a one-way ANOVA or the Kruskal–Wallis test followed by the Bonferroni–Dunn correction. Time course was analyzed by a repeated measures ANOVA or the Friedman test.

Finally, we examined neurological adverse effects in rats following sciatic nerve adhesion. To examine gross motor function, changes in rotarod walking time before and after surgery were compared (Figure 5A). No data are shown for the non-adhesion group because this group comprised the healthy limbs of a randomly selected subset of the total rats used (see Methods). Before surgery, no significant between-group differences in walking time were observed (adhesion, 26.19 ± 6.028; E8002, 27 ± 3.755; E8002 AA(−), 29.11 ± 1.752) (*p* > 0.05). After surgery, both the adhesion group and the E8002 AA(−) group exhibited a non-significant decline in walking time compared to pre-surgery (two weeks: adhesion, 35.42 ± 5.344; E8002, 30 ± 3.049; E8002 AA(−), 24.89 ± 4.562; four weeks: adhesion, 27.86 ± 3.223; E8002, 28.08 ± 4.990; E8002 AA(−), 26.83 ± 7.301; and six weeks: adhesion, 22.03 ± 2.423; E8002, 27.03 ± 3.234; E8002 AA(−), 25.28 ± 4.391 (*p* > 0.05). Crucially, there was no difference in walking time pre- and post-surgery in the E8002 group (*p* > 0.05). Though rotarod walking time was slightly low in the E8002 group and the E8002 AA(−) group at two weeks post-surgery, this may have been due to physical compression of the nerve before the dissolution of the membrane. Indeed, overall, there were no significant between-group differences in rotarod walking time.

As a measure of mechanical sensitivity within the hindlimb, we used a von Frey filament to compare changes in the 50% withdrawal threshold pre- and post-surgery (Figure 5B). Before surgery, no significant between-group differences were observed in the 50% withdrawal threshold of the hindlimb (adhesion, 28.84 ± 0; E8002, 28.84 ± 0; non-adhesion, 28.84 ± 0; E8002 AA(−), 28.84 ± 0) (*p* > 0.05). After surgery, the adhesion group showed a non-significant reduction in the 50% withdrawal threshold (two weeks, 21.82 ± 2.657; four weeks, 26.38 ± 1.197; and six weeks, 24.96 ± 2.118) (*p* < 0.05). Furthermore, no significant between-group differences were observed in the 50% withdrawal threshold of the hindlimbs in the other groups by 6 weeks post-surgery (E8002: two weeks, 22.85 ± 3.312; four weeks, 28.13 ± 0.705; six weeks, 28.84 ± 0; non-adhesion: two weeks, 28.84 ± 0; four weeks, 28.84 ± 0; six weeks, 28.84 ± 0; E8002 AA(−): two weeks, 28.84 ± 0; four weeks, 28.84 ± 0; and six weeks, 28.84 ± 0) (*p* > 0.05). Though the threshold was low in the E8002 group at two weeks post-surgery, the factors and mechanisms involved were unclear.

Overall, our results showed that neurological function was not significantly impaired following sciatic nerve adhesion. Furthermore, none of the rats died during or after surgery, and no obvious adverse effects were observed. Accordingly, the E8002 membrane (with or without AA) had no neurological adverse effects (Figure 5A,B).

### 2.5. l-Ascorbic Acid Enhances Tissue Plasminogen Activator-Mediated Fibrinolysis

**Figure 6:**
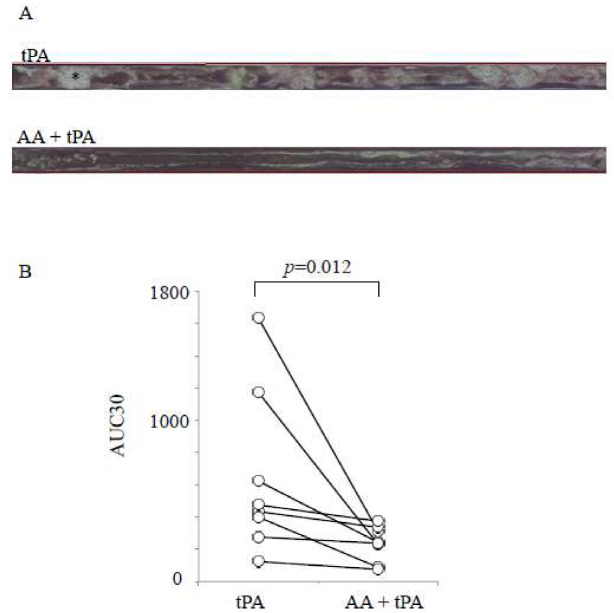
Effect of l-ascorbic acid on recombinant tissue-type plasminogen activator-induced fibrinolysis under flow conditions in human whole blood. (**A**) Representative video microscopy images of fibrinolysis over 18–19 min in samples exposed to recombinant tissue-type plasminogen activator (rtPA) or AA–tPA in human whole blood. The asterisk shows an example of thrombi (white area). (**B**) Area under the curve at 30 min (AUC30) following tPA treatment and AA and tPA treatment in human whole blood (*n* = 8). Overall, the AUC30 was significantly lower following the AA and tPA treatment than the treatment of tPA alone. Values are mean ± SE. Comparisons between two groups were performed using Wilcoxon’s signed rank test.

Our results showed that the AA-containing E8002 membrane was more effective in reducing adhesion formation in our rat sciatic nerve adhesion model than the E8002 membrane without AA (Figure 3 and Figure 4). Therefore, we next investigated whether AA alone could be effective. To this aim, we decided to use human whole blood samples because we ultimately intend to use our treatment approach in human patients. Additionally, we chose to examine tPA-mediated fibrinolysis under flow conditions using the total thrombus-formation analysis system (T-TAS) (Figure 6). The T-TAS is a microchip-based flow chamber system that mimics in vivo conditions and can be used to examine whole blood thrombogenicity. Specifically, the T-TAS was developed for the quantitative analysis of thrombus formation in whole blood specimens from humans [14].

Following the start of perfusion, many small white thrombi adhered to the coated surface of the chamber system. The addition of tPA alone had a limited effect on thrombus formation (Figure 6A). In contrast, in the presence of AA, the frequent collapse of thrombi was detected. The synergistic effect of the AA–tPA combination was determined by calculating the area under the curve at 30 min (AUC30). The AUC30 was significantly lower in the AA–tPA group than the tPA group (*p* = 0.012, *T* = 0; Figure 6B). Thus, AA enhanced tPA-mediated fibrinolysis in human whole blood.

## 3. Discussion

E8002 is a novel multi-layer membrane containing AA that has anti-adhesive effects in both a rat cecal adhesion model and rat laminectomy model [12,13]. Nonetheless, the anti-adhesive effects of E8002 have not been comprehensively examined. Here, we examined E8002 using a sciatic nerve adhesion model to mimic the clinical situation following peripheral nerve surgery. Specifically, we focused on enhancing fibrinolysis by AA and found that E8002 inhibited peripheral nerve adhesions in our rat sciatic nerve model. Corroboratively, we showed that AA enhanced fibrinolysis in human whole blood. Altogether, our results indicated that the AA-containing E8002 membrane has an anti-adhesive effect that may be mediated by fibrinolysis.

Following surgical invasion, the invaded area first fills with platelets and exudate fluid, followed by fibrin deposition [6]. Next, the denuded area is covered by tissue repair cells/macrophages and fibrosis begins. If repair is delayed by decreased fibrinolysis, local inflammation results in adhesion formation.

Creating barriers between the nerve tissue and surrounding tissue is the most effective method to prevent postoperative adhesions. For membrane-like anti-adhesive agents to exert an effect, the following two conditions are required: (1) the damaged surface of all organs must be covered by the anti-adhesive agent until the early fibrin network is formed, and (2) inflammatory cells must not invade the initial fibrin network [1].

Oxidative stress is a key contributor of adhesion formation. Meanwhile, the fibrin-binding affinity of tPA can be impaired by exposure to oxidative stress [8]. Recently, we reported that antioxidants such as uric acid and edaravone enhance fibrinolysis [9,10]. Indeed, in a rat uterine horn model, the intraperitoneal administration of antioxidants (namely AA or vitamin E) effectively prevented postoperative adhesion formation [11]. Though the mechanism of the anti-adhesive effect of antioxidants is unclear, our present study showed that this molecular mechanism might also underlie adhesion formation after peripheral nerve surgery. Given that oxidative stress can induce PAI-1 [15,16], the antioxidant properties of AA may inhibit adhesion formation via the fibrinolytic system, suggesting that fibrinolysis may be an important target for the prevention of adhesions.

**Figure 7:**
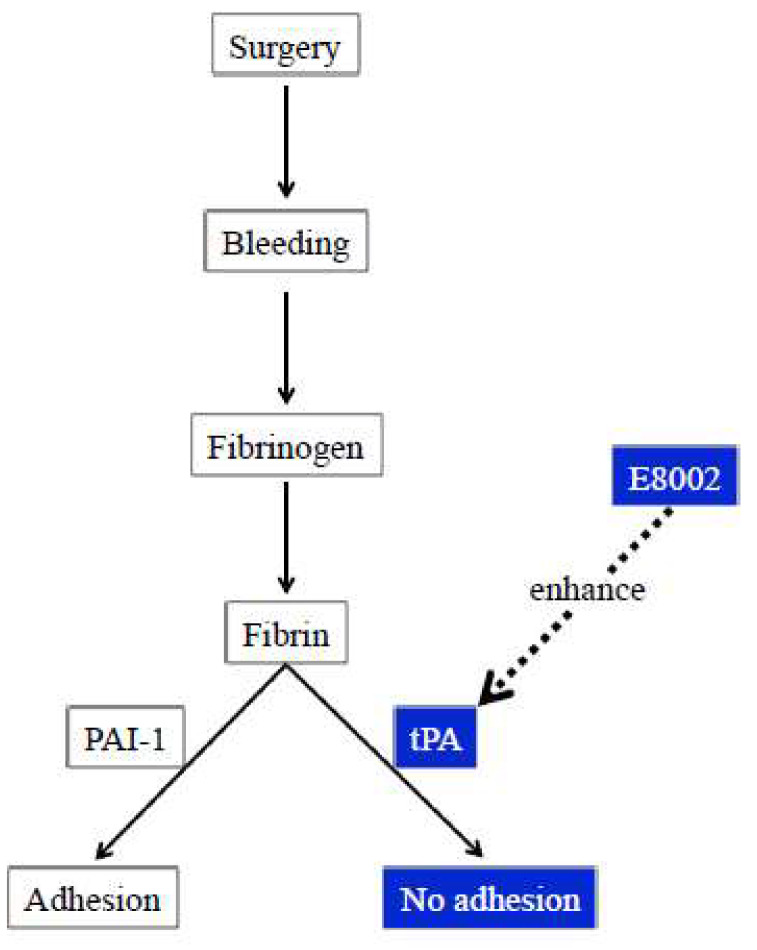
Schema showing the potential anti-adhesive action of l-ascorbic acid-containing E8002 via fibrinolysis. l-ascorbic acid-containing E8002 may exert an anti-adhesive effect by enhancing fibrinolysis. Boxes indicated in blue show mechanisms suggested by the current findings. tPA: tissue plasminogen activator; PAI-1: plasminogen activator inhibitor-1.

These results indicate that the barrier mechanism of E8002 is not enough to induce an anti-adhesion effect and that the activation of fibrinolysis by AA is important. Corroborating this, we found that a higher AA content was associated with a stronger anti-adhesive effect in our cecal model (unpublished data). Accordingly, AA in E8002 may exert anti-adhesion effects via enhanced fibrinolysis (Figure 7). A clinical trial using E8002 is currently ongoing in patients undergoing colostomy and subsequent colostomy closure [17]. However, the spaces surrounding the intra-abdominal organs and those surrounding the peripheral nerve may be different. Nevertheless, even if the types of tissue are different, the molecular targets for adhesion prevention may be the same or at least similar. The results of the ongoing clinical trial could encourage more clinical trials on the use of E8002 in patients scheduled for peripheral nerve surgery.

### Study Limitations

Our study has limitations that should be noted. First, the rotarod and von Frey behavioral tests may not have been relevant readouts for the negative impact of nerve adhesions in this model because our results showed no significant impairment of neurological function following sciatic nerve adhesion. Therefore, a model that causes more adhesions may be necessary to observe an effect. However, we deemed animal models that were more invasive to be unsuitable compared to the respective surgery for patients. Nonetheless, more sensitive behavioral tests would have been more appropriate to show the restorative effects of AA. Second, the effect of AA powder alone was examined in our cecal adhesion animal model, but an anti-adhesion effect was not observed. Therefore, we did not determine the effect of AA alone in our rat model of sciatic nerve adhesion. Third, the effect of oxidative stress in our model of sciatic nerve adhesion should be confirmed. However, the direct measurement of oxidative stress (such as that caused by reactive oxygen species or free radicals) is not straight-forward: reactive oxygen species are activated immediately after treatment and are not likely to be detected in our samples at six weeks post-surgery. Finally, it is also necessary to confirm the effect of tPA and PAI-1 in this sciatic nerve adhesion model. Nonetheless, these enzymes are unfortunately very unstable, and obtaining sufficient reproducibility is problematic because of the circadian variation of endogenous tPA and PAI-1. Additionally, tPA and PAI-1 are also activated immediately after treatment, and any changes are unlikely to be detected at six weeks post-surgery. Therefore, to reveal a direct effect, we examined the enhanced fibrinolytic effect using the T-TAS.

## 4. Materials and Methods

The experimental protocol was approved by the Institutional Animal Care and Use Committee of Kagoshima University (Kagoshima, Japan) (ethical approval number: MD18025; approval date: 10 July 2018). The clinical study protocol was approved by the local ethics committee of Kagoshima University (ethics approval number: 23–115; approval date: 28 December 2011) and carried out following the rules of the Declaration of Helsinki of 1975 (https://www.wma.net/what-we-do/medical-ethics/declaration-of-helsinki/). Written informed consent was obtained from all individuals prior to their participation.

### 4.1. Animals and Experimental Groups

Healthy Sprague–Dawley rats were purchased from Charles River Laboratories Japan, Inc., (Kanagawa, Japan). A total of 21 male rats that were aged 8 weeks and weighed 290–310 g (298.57 ± 1.73 g) were used in this study. All rats were kept under specific pathogen-free housing conditions, and, generally, two animals were housed per cage (with one rat housed in a single cage) in a temperature-controlled room (approximately 23 °C) under a 12 h light–dark cycle. All animals were allowed free access to food and water. Before the evaluation of motor function and mechanical sensitivity, rats were randomly assigned to one of the following three groups: adhesion group (*n* = 7), in which the adhesion procedure was performed but no treatment was subsequently administered; the E8002 AA(−) group (*n* = 7), in which the adhesion procedure was performed followed by nerve wrapping with the E8002 membrane without AA; and the E8002 group (*n* = 7), in which the adhesion procedure was performed followed by nerve wrapping with the AA-containing E8002 membrane. The healthy hindlimbs of seven randomly selected rats from all three groups were classified as the non-adhesion group (*n* = 3, adhesion; *n* = 2, E8002 AA(−); and *n* = 2, E8002 AA(+)). Sham treatment was performed on these seven healthy hindlimbs (see rat sciatic nerve adhesion model). When analyzing results, researchers were blinded to the experimental treatments.

### 4.2. Rat Sciatic Nerve Adhesion Model

A rat sciatic nerve model [1] was used to determine the effects of E8002 on peripheral nerve adhesion. Animals from each group were treated randomly. Anesthesia was induced and maintained with 2.5–3.0% isoflurane inhalation, and the animals were fixed in the lateral position. The procedure was performed in accordance with a previously described sciatic nerve adhesion model [1]. In brief, the left sciatic nerve was carefully exposed and released from the surrounding tissue, including the neural bed, without injury to the nerves or vessels. Scarring and adhesion around the surgically released nerve were produced by repeatedly burning a 10-mm length of the biceps femoris muscle that comprised the neural bed. Burning was performed using a bipolar coagulator (Micro-1D; Mizuho Ika Kogyo Co., Ltd., Tokyo, Japan) to stimulate a local fibrotic response around the sciatic nerve. In the E8002 groups, the E8002 membrane (8 × 4 mm; provided by Kawasumi Laboratories Inc., Tokyo, Japan) was wrapped around the exposed sciatic nerve before suturing. In the sham group, the same surgical procedure was performed but without muscle burning and the wrapping of E8002 around the nerve. The rats’ rectal temperatures (ATB1100; Nihon Kohden, Tokyo, Japan) were monitored during surgery. For postoperative analgesia, all animals were subcutaneously injected with buprenorphine (0.02 mg/kg) twice daily for three days. The extent of scar adhesion and peripheral nerve function were evaluated at 2, 4, and 6 weeks after treatment. The final post-surgical timepoint of 6 weeks was chosen based on our previous experimental report [1].

### 4.3. Assessment of Neurological Adverse Events

Rats were evaluated for motor function [18] and mechanical sensitivity [19] before and 2, 4, and 6 weeks after surgery. Animals were assessed randomly.

For motor function, rats were examined using a rotarod task (MK-670; Muromachi Kikai Co., Ltd., Tokyo, Japan). After acclimatization with the rotarod task, all animals were evaluated. Evaluations were performed by two individuals who were blinded to the group allocation. Each rat was placed on the rotarod cylinder, and the duration the animal remained on the cylinder was measured. The rotation speed increased from 0 to 25 rpm in increments of 2.5 rpm every 6 s. The trial ended if the animal fell off the cylinder. Each animal completed three trials. The longest duration for each animal (in s) was used for the final analysis.

The mechanical sensitivity of the hindlimb was determined by the 50% withdrawal threshold using a von Frey filament (Muromachi Kikai Co., Ltd.). Rats were placed in individual clear plastic cages (12 × 22 cm) on a wire mesh grid (1 × 1 cm) to allow for full access to the ventral aspect of the hind paw. Next, the rats were habituated to the experimental environment (room and apparatus) for approximately 20 min. A logarithmic series of 11 filaments (range: 0.41–28.84 g) was perpendicularly applied to the planter surface of the hind paw until the filament bent. Assessment was initiated with a 3.63 g filament. If the rats withdrew their hind paw, this was counted as an escape response and a thinner filament was used next time. If the rats did not respond, a thicker filament was used next time. The first escape response was noted, and then the process described above was repeated four times using appropriate filaments. The tactile stimulus producing a 50% likelihood of the hind paw withdrawal response (50% withdrawal threshold) was calculated using a top–down formula that has been described previously [20].

### 4.4. Wound Healing Score

The quality of the wound healing of the skin and fascia was examined using a previously described assessment tool [1]. Wound healing scores for the skin and fascia ranged from 1 to 3 (1: skin or muscle fascia entirely closed; 2: skin or muscle fascia partially open; and 3: skin or muscle completely open). Wound healing scores were calculated for each rat. Evaluations were performed by two individuals who were blinded to the group allocation.

### 4.5. Nerve Adhesion Score

Nerve adhesions were examined using a previously described assessment tool [1]. In brief: grade 1 denotes that the nerve was either free or required minimal blunt dissection to separate, grade 2 denotes that moderate or vigorous blunt dissection was needed to separate the nerve, and grade 3 denotes that sharp dissection with scissors was required for nerve separation. Nerve adhesion scores ranged from 1 to 3 (1: no dissection or mild blunt dissection required; 2: some vigorous blunt dissection required; and 3: sharp dissection required during neurolysis to the area of adhesion). Adhesion scores were calculated for each rat. If no surgery has been performed, the adhesion score would be expected to be 1. Evaluations were performed by two individuals who were blinded to the group allocation.

### 4.6. Histological Examination of Adhesions

Six weeks after surgery, the rats were deeply anesthetized by the intraperitoneal injection of pentobarbital sodium (100 mg/kg), and perfused with heparin physiological saline, followed by 4% paraformaldehyde in a 0.1 M phosphate buffer (pH 7.4) via the heart.

The sciatic nerve and surrounding soft tissue (including the neural bed) were harvested. Samples were fixed in 4% paraformaldehyde at 4 °C overnight and decalcified in Kalkitox (Wako Pure Chemical Industries Ltd., Osaka, Japan) at 4 °C for 2 days. Next, samples were immersed in a 5% sodium sulfate solution at 4 °C overnight, dehydrated in a graded ethanol series, cleared with xylene, and embedded in paraffin.

To identify connective tissues, such as elastic fibers and collagen fibers, aldehyde fuchsin Masson–Goldner staining was performed. The degree of fibrous adhesion formation was examined using a light microscope at 400× magnification (DP21; Olympus Optical Co., Tokyo, Japan). To quantify the sciatic nerve adhesion site, random fields from the region between nerve tissue and muscle tissue of interest were imaged at 40× magnification (360 × 270 μm). Next, the ratio of the area covered by light blue/green was determined using Image J (National Institutes of Health, Bethesda, MD, USA). The optical density ratio was calculated based on the optical density of the area selected from the non-adhesion group.

### 4.7. Human Blood Samples

Blood samples from eight healthy, fasting Japanese volunteers (6 men and 2 women with a mean age of 41.50 ± 13.53 years) were collected, as previously described [9,10]. The body mass indexes of the participants were unknown, but their general health was good. None of the volunteers had taken drugs, including antithrombotic drugs, within two weeks of the study. Blood samples were collected from 9 a.m. to 11 a.m. in plastic tubes containing 3.2% sodium citrate (Terumo Co., Tokyo, Japan).

### 4.8. Assessment of Fibrinolysis Using the Total Thrombus-Formation Analysis System

Normal ranges for the analysis of the T-TAS have not yet been defined, but T-TAS findings for all volunteers’ samples were within 95% of the median of 123 healthy Japanese individuals who participated in a preliminary study (data not shown).

The thrombolytic effects of the combination of AA and recombinant tissue-type plasminogen activator (tPA; alteplase) were compared to tPA alone under flow conditions using the T-TAS^®^ (Fujimori Kogyo Co., Ltd., Tokyo, Japan) in whole blood, as previously described [9,10]. AA was added to blood samples 10 min before the addition of tPA. Once tPA was administered, each sample was perfused over a microchip coated with collagen and tissue thromboplastin to promote thrombosis at a flow rate of 4 μL/min (corresponding to an initial wall shear rate of 240/s). Thrombogenesis and thrombolysis were observed in the microchip using a built-in light microscope. Based on our previous reports, 500 IU/mL of tPA was selected as the final concentration [9,10]. The selection of the final concentration of AA (6 µM; obtained from Kanto Kagaku Co., Ltd., Tokyo, Japan) was based on a preliminary experiment (data not shown).

### 4.9. Statistical Analysis

Statistical analyses were performed with either parametric or non-parametric tests after the Shapiro Wilk test. The Kruskal–Wallis test was used to examine wound healing and adhesion scores. An ANOVA or the Kruskal–Wallis test followed by the Bonferroni–Dunn correction for multiple comparisons were used for between-group analyses of rotarod walking time and the 50% withdrawal threshold. The time course for the 50% withdrawal threshold and rotarod walking time were analyzed using a repeated measures ANOVA or the Friedman test. The optical density of the adhesion sites was examined using a one-way ANOVA followed by the Bonferroni–Dunn correction for multiple comparisons. The AUC30 was calculated to determine the extent of thrombolysis, as previously described [9,10]. Comparisons between two groups were performed using Wilcoxon’s signed rank test. Data are expressed as mean ± standard error (SE). *p* Values < 0.05 were considered statistically significant. All data were analyzed using SPSS version 24 (IBM, Chicago, IL, USA).

## 5. Conclusions

E8002 containing AA inhibited the formation of peripheral nerve adhesions in a rat sciatic nerve adhesion model. Furthermore, in human whole blood samples, AA enhanced tPA-mediated fibrinolysis, potentially by preventing oxidative stress, which, in turn, may have inhibited fibrinolysis in thrombi via tPA. The outcome of future clinical studies will help determine whether E8002 is an innovative preventive treatment for peripheral nerve adhesion.
